# Chronic Pulmonary Aspergillosis in Patients with Underlying Respiratory Disorders in Cuba—A Pilot Study

**DOI:** 10.3390/jof5010018

**Published:** 2019-02-22

**Authors:** Nathalie Beltrán Rodríguez, Javier Luis San Juan-Galán, Carlos Manuel Fernández Andreu, Dulce María Yera, Miriam Barrios Pita, Mayda Rosa Perurena Lancha, Rosario Esperanza Velar Martínez, María Teresa Illnait Zaragozí, Gerardo Félix Martínez Machín

**Affiliations:** 1 Benéfico-Jurídico Pneumological Hospital, 10600 Havana, Cuba; nathaliebeltran@infomed.sld.cu (N.B.R.); bibliobecjuridico@infomed.sld.cu (D.M.Y.); mirita@infomed.sld.cu (M.B.P.); 2 Pedro Kourí Institute of Tropical Medicine, 17100 Havana, Cuba; jlsanjuan9@ipk.sld.cu (J.L.S.J.-G.); cfandreu@ipk.sld.cu (C.M.F.A.); mrpl@ipk.sld.cu (M.R.P.L.); velar@ipk.sld.cu (R.E.V.M.); mtilnait@ipk.sld.cu (M.T.I.Z.)

**Keywords:** aspergillosis, diagnosis, cavities, IgG, itraconazole

## Abstract

Chronic pulmonary aspergillosis (CPA) is a fungal infection with high mortality and morbidity rates. This disease is caused by several *Aspergillus* species and affects patients with an underlying respiratory condition. This pilot study aims to recognize CPA among patients with different respiratory diseases. Twenty-one out of 47 patients were classified as CPA based on the examination of clinical signs and symptoms, radiological findings, mycological culture of respiratory samples and analysis of *Aspergillus* IgG antibodies. There was a close association between high levels of *Aspergillus* IgG antibodies and the presence of cavities. Although *Aspergillus flavus* was the predominant species among clinical isolates, the number of isolates was small to reach conclusions on the prevalence of this species as main cause of CPA in Cuba. From the eleven evaluable patients for the treatment with itraconazole (Lozartil^®^), nine improved their health status while two did not show any recovery. This drug is included in the therapy schemes for aspergillosis in Cuba.

## 1. Introduction

Chronic pulmonary aspergillosis (CPA) is a fungal infection that causes the death of more than 450,000 patients around the world every year and the estimated morbidity rate is more than 3,000,000 patients per year [1]. The aetiological agents of this disease are different *Aspergillus* species (mainly *A. fumigatus*) which are opportunistic fungi widespread in the aeromycobiota and are very well adapted to extreme changes of the environment [2].

Patients with CPA are commonly non-immunocompromised individuals and must have an underlying lung condition such as cavities, chronic obstructive pulmonary disease (COPD), bullous lung disease after or through pulmonary tuberculosis (PTB), undetermined bronchiectasis or pneumothorax [3]. In Cuba, there are high indexes of COPD (~39.5 per 100,000), lung cancer (~58.3 per 100,000) and tabaquism (~19,676.3 per 100,000) and although there is a National Program for the Control of PTB, there are new cases of the disease every year (~5.9 per 100,000) [4,5].

Because of the clinical likeness of CPA with other respiratory diseases, it is not rare to misdiagnose it as other mycoses or bacterial infections. The diagnosis of CPA is an integrated process where the clinicians must analyze clinical features of the patient, radiological images and lab results from direct exam, culture and serology [6]. This is a preliminary study to evaluate CPA in patients with underlying respiratory conditions through clinical examination, analysis of radiological images and microbiological/ serological tests in Cuba.

## 2. Materials and Methods

### 2.1. Patients

From September, 2017 to April, 2018, 47 patients at Benéfico-Jurídico Pneumological Hospital (BJPH) were included in the study. The sample had 24 men and 23 women with an average age of 62.21 years and 47.59 years respectively. The selection was carried out using the CPA definition of the European Society for Clinical Microbiology and Infectious Diseases and European Respiratory Society [6]. The inclusion criteria were non-immunocompromised patients with: (i) clinical signs and symptoms such as productive cough, weight loss, bronchiectasis or hemoptysis sustained for at least three months; (ii) radiological findings suggesting any CPA features such as cavitary pulmonary lesions, paracavitary infiltrates, pleural thickening and nodules; (iii) an underlying respiratory condition related or not to a known disease such as idiopathic bronchiectasis, COPD or previous PTB cases. Exclusion criteria were: (i) patients with previous antifungal treatment in the last two months before the clinical review and (ii) patients with asthma or other disease with a similar presentation (active PTB, endemic mycoses, Wegener’s granulomatosis). Patients enrolled in the study signed an informative consent before performing any test and the protocol was approved by the Ethical Committees of the BJPH and Pedro Kourí Institute of Tropical Medicine in accordance with the Declaration of Helsinki (Project Identification Code: CEI-IPK 06-18). Every CPA case was defined on the recognition of all inclusion and exclusion criteria and a positive result for *Aspergillus* IgG antibodies. Positive culture for *Aspergillus* sp. from a respiratory sample other than sputum was taken as strong evidence. Results from sputum cultures were only taken into consideration for the case diagnosis when it was three times positive.

### 2.2. Clinical Samples

Sputa, bronchoalveolar lavages or pleural infiltrates were selected as possible respiratory specimens from patients. The samples were inoculated in Sabouraud dextrose agar supplemented with chloramphenicol (0.5%) for 7–10 days at 28 °C and 37 °C.

### 2.3. Isolates Identification

The identification of the recovered isolates was based only on morphological characteristics. The flow diagram used for identification of species was the one recommended by Samson et al. [7]; briefly: *Aspergillus* isolates recovered from respiratory samples were sub-cultured for 7 days at 25 °C and 50 °C in Czapek yeast extract agar (CYA) and in malt extract agar (MEA). Macromorphology and micromorphology were compared, following the taxonomical key described by de Hoog et al. [8].

### 2.4. Serology

IgG *Aspergillus* antibodies were determined in serum samples from every patient using *Aspergillus fumigatus* IgG ELISA (IBL-America) and the readings were made in ELISA Reader ELx808, Bio-Tek. As stated by the manufacturer, definitions for negative, equivocal and positive results were applied according to the titer levels (negative: < 8 U/mL; equivocal: 8–12 U/mL; positive: > 12 U/mL).

### 2.5. Treatment

Patients with confirmed diagnosis of CPA were treated with 400 mg/day of itraconazole (Lozartil^®^, Novag) for at least six months as it is the standard of care for this antifungal in Cuba.

### 2.6. Statistical Analysis

The statistical description of the variables was performed, including frequency tables. Also, a Fisher’s exact test was performed to determine the association between bronchiectasis, COPD, cavities and serology results (negative or positive). Every test was performed using GraphPad Prism v.6.01 and differences were considered significant at a *p* value of <0.05.

## 3. Results

At the end of the study out of 47 patients 21 of them were classified as CPA, following all clinical and radiological criteria as well as the positive results for the IgG test. Male were prevalent within positive cases with 57.14% (12/21) and the average age among patients was 54 years. Table 1 summarizes comorbidities associated to patients with CPA.

**Figure 1:**
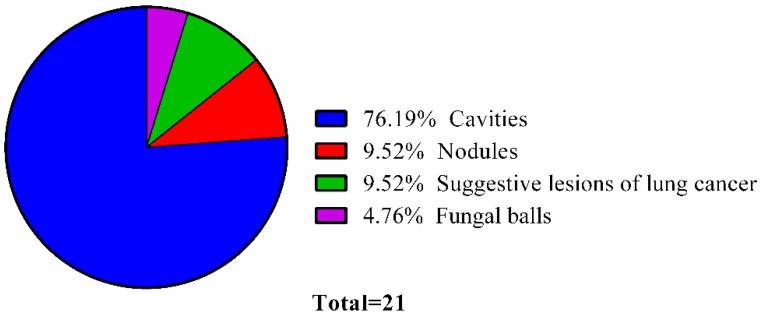
Radiological findings observed in the patients with CPA. (*n* = 21).

Table 2 shows the clinical signs and symptoms identified in the patients with CPA. The radiological recognition revealed images of cavities, nodules and lesions that were suggestive of lung cancer without confirmation and fungal balls. Figure 1 exhibits the distribution of the radiological findings.

The Fisher’s exact test showed no relationship between COPD or idiopathic bronchiectasis with the serology result (*p* = 0.746 and *p* = 0.528 respectively), however it was significant for cavities and the serology result with an odd-ratio = 7.2 (*p* = 0.003).

Five cultures were positive, corresponding to three isolates of *Aspergillus flavus*, one isolate of *A. fumigatus* and one isolate of *A. niger*.

Of the 21 patients receiving treatment with itraconazole, only eleven were evaluable. Nine patients improved their clinical status and their radiological features while two of them did not show any recovery.

## 4. Discussion

Chronic pulmonary aspergillosis is a very complex pulmonary syndrome that represents a challenge both for diagnosis and treatment. In our study, most of the patients were middle-age persons and there was a minor difference in the presentation of CPA in gender, with a predominance in men. These results are similar to other investigations [9,10,11] and according to experts the disease is more frequent in middle-aged men [12]. Aging affects the anatomy of the lungs, degenerating lung parenchyma and the elastic fibers around the alveolar duct. This process starts at the age of 50 and results in expansion of airspaces and reduction in supporting tissue, so, over time, the risk of acquiring a chronic infectious/non-infectious disease increases [13].

Having an underlying respiratory condition is a risk factor for the development of CPA. As part of the study we tested the association between serology results and a respiratory morbidity (COPD and idiopathic bronchiectasis), yet there was no association between these conditions, which does not mean that patients with these characteristics are “safe groups”. Several studies report that airway colonization by *Aspergillus* species could be a threat for the development of aspergillosis after contracting COPD, where there is poor lung function and the use of corticosteroids is usual [14,15,16,17]. Cuban health stats from 2017 report that chronic diseases of the lower airways rank sixth place in frequency among mortality causes in the country. Moreover, in 2017 the incidence of pulmonary tuberculosis per 100,000 population within 15-64 years was 5.9, slightly higher than the incidence in the previous year (5.6 per 100,000) [5]. Still, Cuba is considered a country with low incidence of PTB in the American region according to WHO reports [18]. Estimates of CPA incidence and prevalence based on this data could help to understand the magnitude of this health issue.

Despite excluding patients with active PTB from the study, at the end of processing the data one of the patients diagnosed with CPA was also positive by GeneXpert for PTB. The fact is that *Aspergillus* and *Mycobacterium tuberculosis* are ecologically “related” because the first one takes advantage of the lung damage caused by the last. Hedayati et al. report 13.7% of patients with CPA are co-infected with *M. tuberculosis* [10]. In view of this we decided to include this case in the results and we suggest that patients with PTB should be tested for *Aspergillus* IgG antibodies just for discarding the possibility of a co-infection.

Interestingly, there was a large group of patients without previous diagnostic conclusion that meet all the clinical, radiological and serological criteria for CPA. Most of these patients were previously treated for bacterial infections, including pulmonary tuberculosis, without improvement. CPA have a clinical presentation similar to other respiratory diseases, which is why it is so difficult to diagnose. Although the clinical assistance in Cuba is remarkable, there are very few reports of this disease in the country and for that matter CPA is poorly suspected and confused with other chronic diseases such as PTB.

Our study revealed that productive cough was the most common symptom among these patients with CPA, but this is not a reliable marker for the diagnosis since most of patients with an infectious respiratory disease have it. In relation to the case definition, signs and symptoms might be unspecific except for hemoptysis and chest pain. Weight loss also can be an indicative symptom of CPA although is not a defining one. More important, the duration of signs and symptoms must be over 3 months to assert the chronicity of the disease [19].

The prevalence of cavities as the main radiological finding also showed a statistical significance, establishing 7.2 times more probability to have a positive serology result in these patients than in those without cavities. Maghrabi and Denning report that cavities and pleural thickening are the commonest radiological feature in their patients with CPA at the United Kingdom National Aspergillosis Centre in Manchester [20]. Cavities are usually thick-walled and are located in the upper lobes of the lung but this is not an exclusive feature of CPA since there are diseases like pulmonary tuberculosis, lung cancer and other mycoses that are very similar [21,22]. Computed tomogram (CT) can be a very useful tool for recognition, management and follow-up of aspergillosis cases, providing more evidence on the characteristics of the lesions than traditional chest X-ray radiography [23].

Detection of high IgG levels against *Aspergillus* is a heavy evidence of infection and may help to distinguish CPA from colonization or other diseases. Most of the commercial kits for detection of anti-*Aspergillus* IgG display specificity and sensitivity over 80% and are recognized as the gold standard tests for the diagnosis [24]. However, patients with chronic lung diseases or other clinical forms of aspergillosis like allergic bronchopulmonary aspergillosis may have raised levels of *Aspergillus* IgG antibodies and therefore be detected as false positive cases. Page et al. recommend to adjust the cutoff values of commercial kits in order to reduce this risk and increase the certainty of a positive case. The *Aspergillus* IgG antibodies test from Genesis Diagnostics Ltd. has very similar parameters compared to the kit we used in our study and it was proven to be more specific when 20 U/mL was used as the cutoff value [25]. For future studies we should optimize the kit used in our population and under our conditions.

There are some clinical presentations such as *Aspergillus* nodules or early stages of the disease that can exhibit IgG levels below the positive threshold of the test and therefore could be misdiagnosed as false negatives [9]. Furthermore, CPA can be caused by non-*A. fumigatus* species which may not be detected by indirect serological methods specific for *A. fumigatus* though some antigens are shared among species and the tests can showed cross-reactivity [26]. Due to this it is probable that a few cases were excluded as false negative cases during the selection.

When culture, bacilloscopy and/or molecular tests are negative in a presumed case of PTB, clinicians must consider a CPA diagnosis. Gbaja-Biamila et al. recommend mycological study of smear-negative pulmonary tuberculosis to discard the possibility of a CPA, with *Aspergillus* IgG assay as an essential test [27]. According to Denning et al. the only way to identify CPA in patients with residual pulmonary shadows of pulmonary tuberculosis is through microbiological testing with special emphasis on *Aspergillus* IgG antibodies [28].

The positivity rate for culture was very low, but these results could be due to most of our clinical samples being sputa. As stated by the Infectious Disease Society of America, the mycological culture of sputum has a low frequency of recovery [29]. Even positive sputum cultures are questionable since *Aspergillus* is widespread in the environment and can be part of mycobiota of the oral cavity and the upper airways [30]. In addition, there are other microorganisms in the oral microbiota that grow faster than *Aspergillus* and might hinder the result of the culture. Pashley et al. try to reduce these problems by increasing the amount of sample to process, extending the incubation period to seven days, applying mucolytic agents to process the sputa and using potato dextrose agar supplemented with gentamicin, cloramphenicol and fluconazole for mycological culture, all this with significantly higher recovery results for *A. fumigatus* than the standardized procedure recommended by the UK Health Protection Agency [31]. This methodology should be considered to improve the isolation of *Aspergillus* species from respiratory specimens, specifically sputum.

Although *A. fumigatus* is the more relevant species in CPA, other species like *A. flavus* and *A. niger* also can be found causing this infection and are a significant cause of pulmonary aspergillosis in countries similar in geography and climatic conditions to Cuba [32,33]. Many studies of indoor/outdoor mycobiota in Havana reveal *A. flavus* and *A. niger* as predominant, over *A. fumigatus* [34,35,36]. However, the number of isolates recovered was insufficient to make conclusions about the possibility of defining *A. flavus* or *A. niger* as a major cause of CPA. Further investigations must be done to estimate the prevalence of these other species as etiologic agents of aspergillosis in Cuba.

Besides macro and micromorphology differences are well defined for the most frequent *Aspergillus* species, growth parameters can be very useful during the identification. For example, *A. fumigatus* produces smaller colonies than *A. flavus* under the same incubation conditions. However, if the temperature is risen up to ≥40 °C, *A. fumigatus* germination is enhanced while *A. flavus* germination decay. Also pH and albumin concentration can affect the germination process among species [37].

According to the main international guidelines for the treatment of chronic forms of aspergillosis, voriconazole is the first line in antifungal therapy while itraconazole can be used as an alternative. Since CPA is a chronic infection the dosage recommended for these cases requires long terms and recurrent liver checking [6,29]. From the evaluable patients there was a considerable number that improved their health condition by being under treatment with itraconazole. This fact also works as a diagnosis criterion, emphasizing the reliability of the protocol designed for the present study. Unfortunately, there were two patients who did not recover and we could not know if this was due to low drug absorption or if the patients were infected with itraconazole resistant isolates. Also these two cases could have been false positives. The use of media culture with itraconazole for the isolation of *Aspergillus* from clinical samples can be a complementary test for the initial screening of antifungal resistance, yet antifungal susceptibility testing should performed to obtain more conclusive data about the minimum inhibitory concentration. Study limitations must be considered for treatment results as it was performed by qualitative methods involving the resolution of the clinical symptomatology and the stability of radiological images. There are quantitative parameters like body mass index and results from functional respiratory tests which are more informative for the treatment evaluation, nevertheless, this is a preliminary study and these aspects will be assured for future studies. While voriconazole is not imported into our country, itraconazole can be a suitable option for the management of aspergillosis in Cuba.

## 5. Conclusions

The schemes for the diagnosis of respiratory diseases often underestimate fungal infections, especially CPA, because the appropriate tests are not performed. Clinicians must be aware of radiological and clinical signs of this disease and should look for support in microbiological tests, particularly for IgG antibodies detection. Also, the correct identification of CPA would modify national health statistics on tuberculosis and other diseases and could improve life expectancy for these patients and save resources from a mistaken diagnosis.

## Figures and Tables

**Table 1:** Co-morbidities associated to CPA in the studied patients. (*n* = 21).

Co-Morbidities	No. of Patients (%)
COPD	4 (19.05)
Lung cancer	4 (19.05)
Idiopathic bronchiectasis	5 (23.81)
Pleural abscess	1 (4.76)
PTB	1 (4.76)
Without diagnostic conclusion	6 (28.57)

**Table 2:** Clinical signs and symptoms observed in patients diagnosed with CPA. (*n* = 21).

Clinical Signs and Symptoms	No. of Patients (%)
Productive cough	21 (100)
Dyspnea	9 (42.86)
Hemoptysis	5 (23.81)
Chest discomfort	5 (23.81)
Weight loss	5 (23.81)
Tiredness	3 (14.29)
Anorexia	2 (9.52)
